# Supplementary material for: E2F1 Promotes Progression of Bladder Cancer by Modulating RAD54L Involved in Homologous Recombination Repair
Source: Int J Mol Sci. 2020 Nov 27;21(23):9025. doi: 10.3390/ijms21239025 (PMC7730422; doi:10.3390/ijms21239025)
Supplement: Supplementary file 1 [file ijms-21-09025-s001.pdf]

A

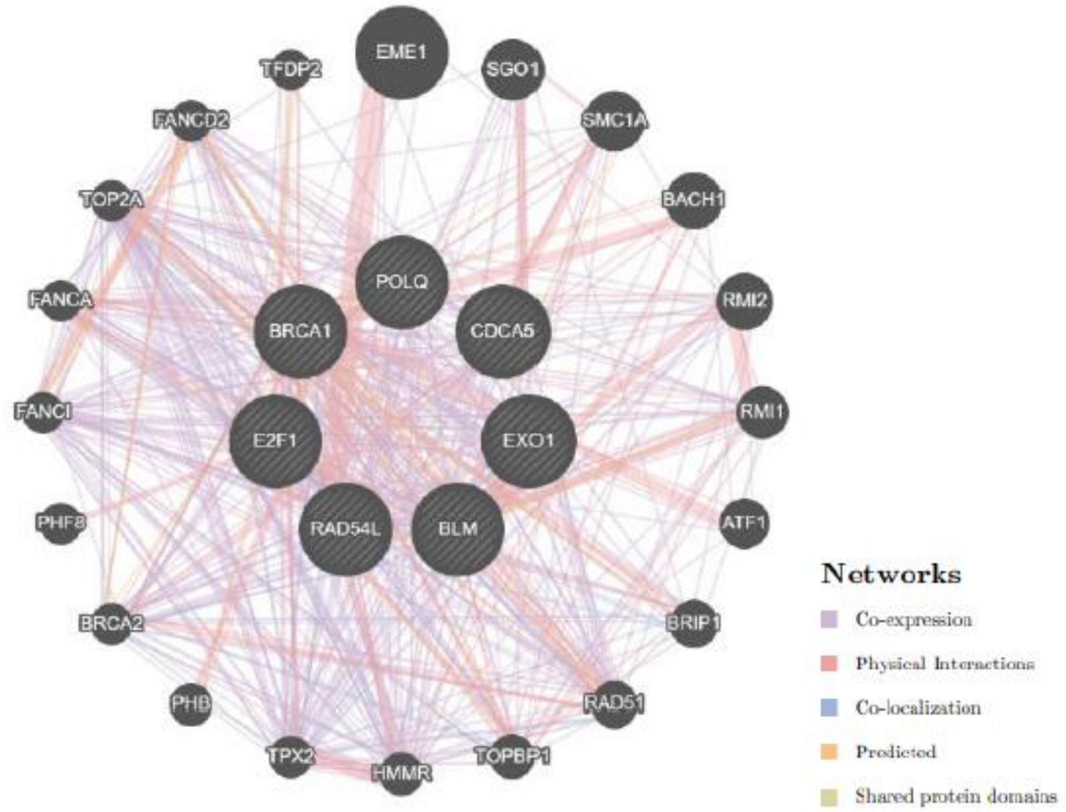

B

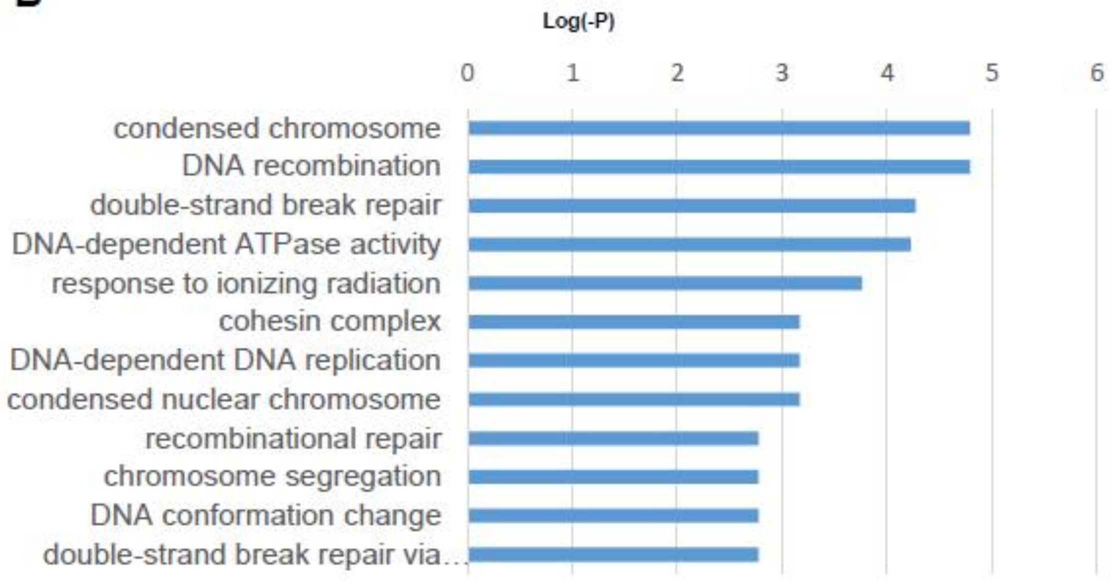

Figure S1 Gene-gene network analysis showing E2F1 correlated genes. (**A**) Gene-gene interaction network associated with DNA repair genes. Each colored line represents functional and physical interactions in the literature, respectively. These networks were generated by GeneMANIA (http://genemania.org) **(B)** GeneMANIA function analysis and significant enrichment of selected genes in bladder cancer patients.

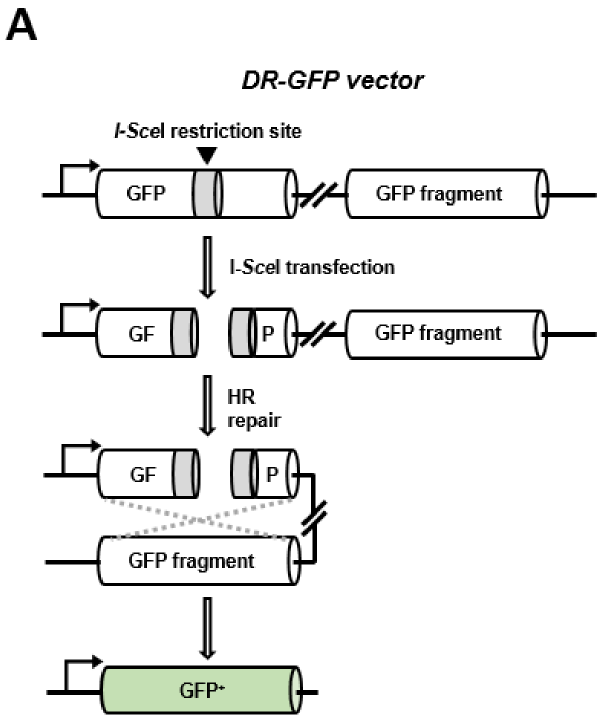

Figure S2 Schematic diagram of HR assay by DR-GFP. (**A**) DR-GFP vector contains an I-SceI endonuclease site within the coding region, which abolishes GFP expression. Expression of I-SceI induces single DSBs.

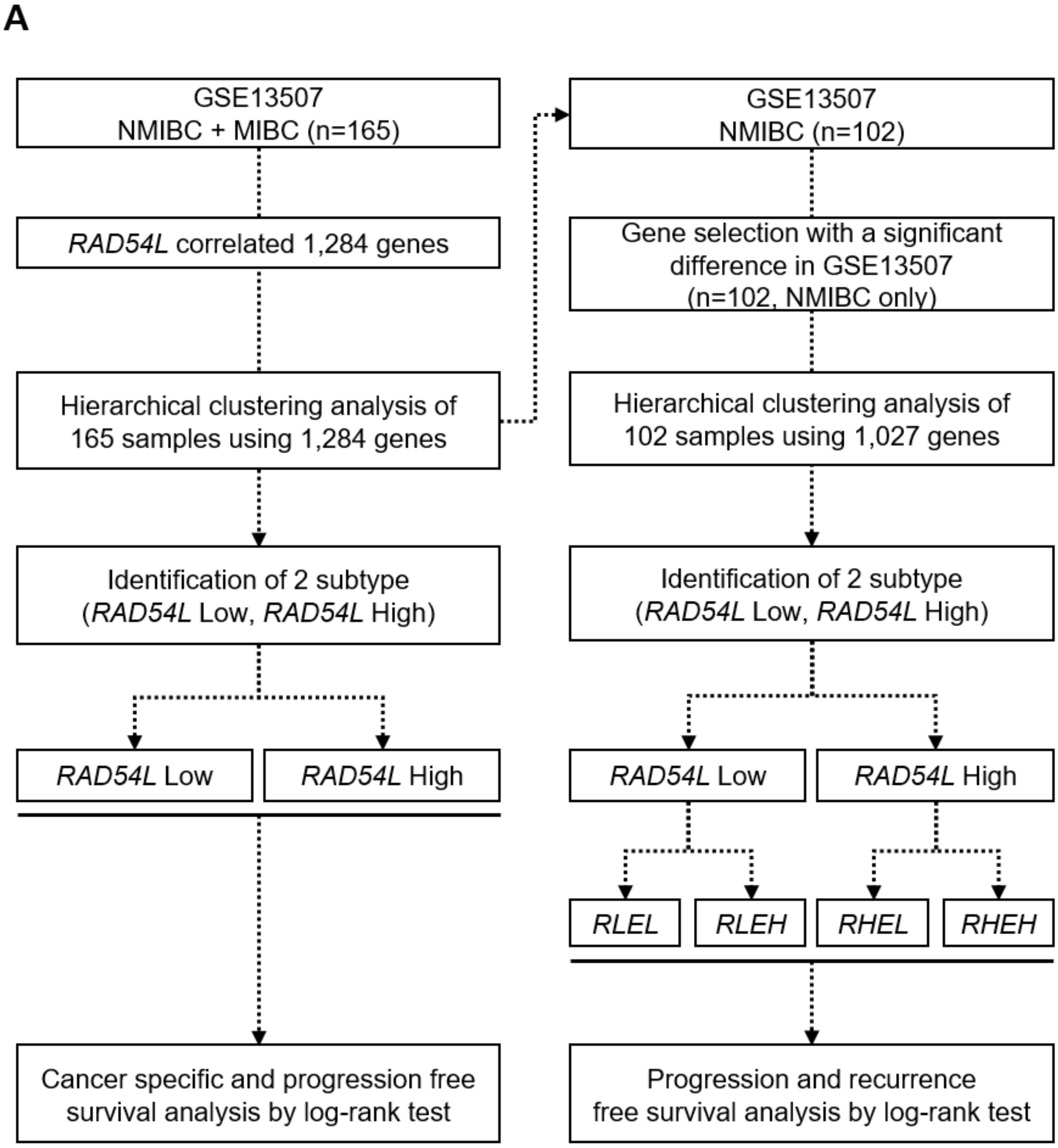

Figure S3 Schematic diagram of bioinformatics. (**A**) RLEL, RAD54L low and E2F1 low; RLEH, RAD54L low and E2F1 high; RHEL, RAD54L high and E2F1 low; RHEH, RAD54L high and E2F1 high.

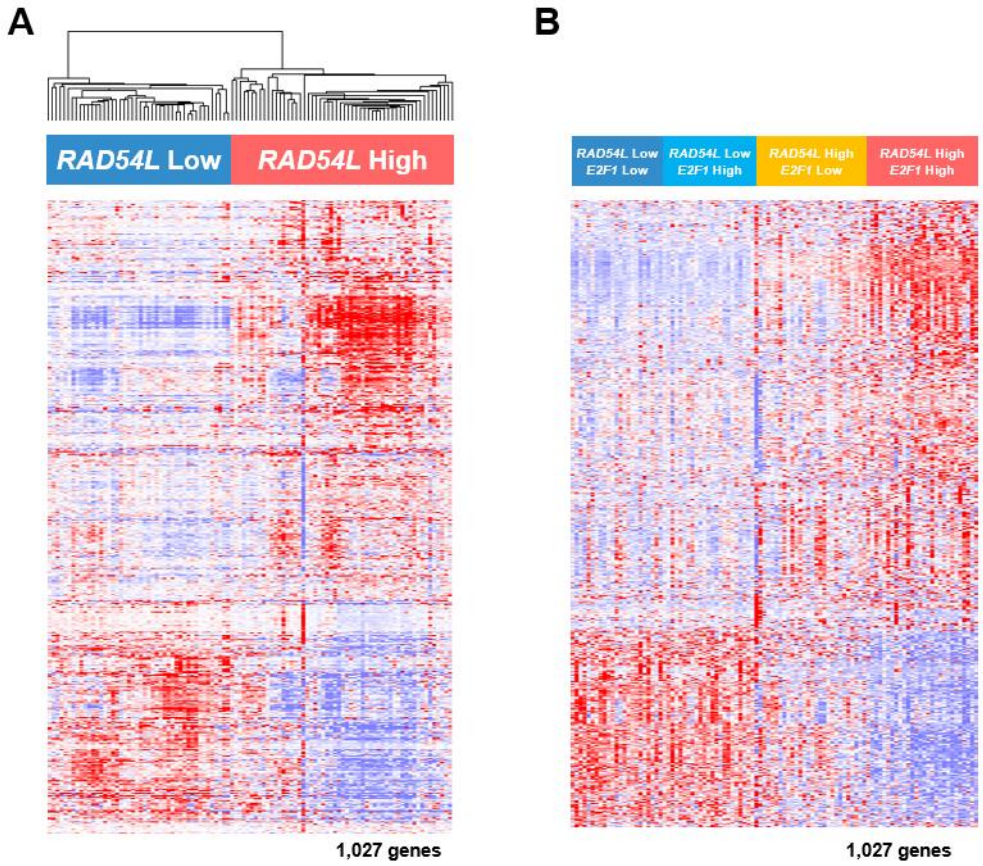

Figure S4 Hierarchical clustering analysis of gene expression data from GSE13507 for patients with NMIBC. (**A**) Heat map of RAD54L correlated 1,027 genes in 102 NMIBC patients. Two subtypes of patients were revealed from unsupervised clustering analysis using RAD54L correlated 1,027 genes. (**B**) Depending on the expression of E2F1, two subtypes are redefined into four 4 new subtypes.

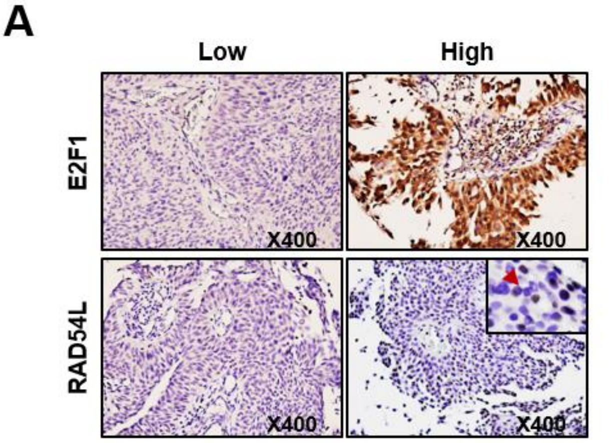

E2F1	RAD54L	Non-recur N=11	Recur N=17
Low	Low	2 (18.2)	0 (0)
Low	High	0 (0)	0 (0)
High	Low	1 (9.1)	6 (35.3)
High	High	8 (72.7)	11 (64.7)

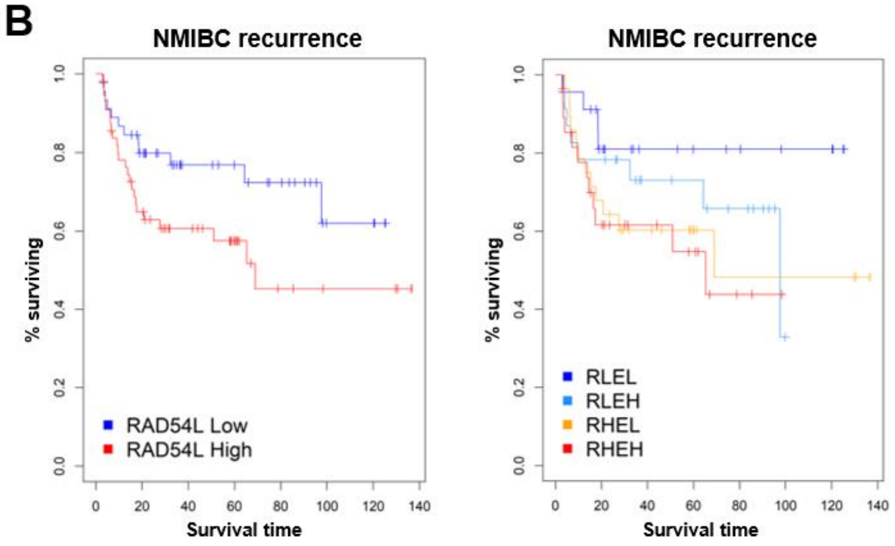

	# of Sample	Observed	Expected
RAD54L Low	46	12	17.754
RAD54L High	56	24	18.246
Result	Chisq = 3.721	df = 1	p = 0.05

	# of Sample	Observed	Expected
RLEL	23	4	8.918
RLEH	23	8	8.835
RHEL	28	12	9.867
RHEH	28	12	8.379
Result	Chisq = 4.868	df = 3	p = 0.18

Figure S5 E2F1 and RAD54L expression levels are not correlated with recurrence in BLC patients. (**A**) IHC staining with anti-E2F1, RAD54L antibody was performed for non-recurrent BLC tumor tissues and recurrent BLC tumor tissues. Photographs were taken at a magnification of 400×. (**B**) Recurrence-free survival in NMIBC patients (GSE13507, n = 102, p = 0.05 by log-rank test, left panel). Recurrence-free survival in NMIBC patients (GSE13507, n = 102, p = 0.18 by log-rank test, right panel). RLEL, RAD54L low and E2F1 low; RLEH, RAD54L low and E2F1 high; RHEL, RAD54L high and E2F1 low; RHEH, RAD54L high and E2F1 high.
